# Supplementary material for: The mediating effect of platform width on the size and shape of stone flakes
Source: PLoS One. 2022 Jan 21;17(1):e0262920. doi: 10.1371/journal.pone.0262920 (PMC8782408; doi:10.1371/journal.pone.0262920)
Supplement: S1 Table — (PDF) [file pone.0262920.s001.pdf]

Supporting Information 1 Tables

SI Table 1a. Linear models used for the mediation analysis on flake weight among the glass flake assemblage. PD is transformed by cube-root and PW by square-root to achieve an approximately symmetrical distribution.

**Full model**: A Poisson linear model summarizing the effect of PD, PW and EPA on flake weight among the glass flake assemblage.				
	Est. coef.	Std. error	z value	p
Intercept	2.10	0.03	62.69	<.001
PD	0.63	0.07	9.59	<.001
PW	0.47	0.06	7.24	<.001
EPA	0.58	0.04	16.12	<.001
Pearson-based dispersion value=1.08.<br>Likelihood ratio test: deviance=983.29, df=3, p<.001.<br>Variance Inflation Factor: PD=6.41, PW=5.93, EPA=2.33.				
**Mediator model**: A Gaussian linear model summarizing the effect of PD and EPA on PW among the glass flake assemblage.				
	Est. coef.	Std. error	t value	p
Intercept	<0.0001	0.03	0.00	>0.99
PD	0.94	0.03	27.45	<.001
EPA	0.02	0.03	0.59	0.55
$F(2\|147)=470.7$, p<.001; Adjusted R^2=0.86.<br>Variance Inflation Factor: PD=1.27, EPA=1.27.				

SI Table 1b. Linear models used for the mediation analysis on flake length among the glass flake assemblage. PD is transformed by cube-root and PW by square-root to achieve an approximately symmetrical distribution.

**Full model**: A Gaussian linear model summarizing the effect of PD, PW and EPA on flake length among the glass flake assemblage.				
	Est. coef.	Std. error	z value	p
Intercept	65.89	1.27	51.97	<.001
PD	20.18	3.56	5.68	<.001
PW	-1.48	3.46	-0.43	0.67
EPA	19.82	1.44	13.78	<.001
$F(3\|146)=82.31$, p<.001; Adjusted R^2=0.62.<br>Variance Inflation Factor: PD=7.81, PW=7.40, EPA=1.28.				
**Mediator model**: A Gaussian linear model summarizing the effect of PD and EPA on PW among the glass flake assemblage.				
	Est. coef.	Std. error	z value	p
Intercept	<0.0001	0.03	0.00	>0.99
PD	0.94	0.03	27.45	<.001
EPA	0.02	0.03	0.59	0.55

F(2|147)=470.7, p<.001; Adjusted R^2=0.86.
Variance Inflation Factor: PD=1.27, EPA=1.27.

SI Table 1c. Linear models used for the mediation analysis on flake width among the glass flake assemblage. PD is transformed by cube-root and PW by square-root to achieve an approximately symmetrical distribution.

Full model: A Gaussian linear model summarizing the effect of PD, PW and EPA on flake width among the glass flake assemblage.

	Est. coef.	Std. error	z value	p
Intercept	25.51	0.46	55.28	<.001
PD	-3.11	1.29	-2.40	0.02
PW	10.36	1.26	8.22	<.001
EPA	1.89	0.52	3.61	<.001

F(3|146)=77.36, p<.001; Adjusted R^2=0.61.
Variance Inflation Factor: PD=7.81, PW=7.40, EPA=1.28.

Mediator model: A Gaussian linear model summarizing the effect of PD and EPA on PW among the glass flake assemblage.

	Est. coef.	Std. error	z value	p
Intercept	<0.001	0.03	0.00	>0.99
PD	0.94	0.03	27.45	<.001
EPA	0.02	0.03	0.59	0.55

F(2|147)=470.7, p<.001; Adjusted R^2=0.86.
Variance Inflation Factor: PD=1.27, EPA=1.27.

SI Table 1d. Linear models used for the mediation analysis on flake thickness among the glass flake assemblage. PD is transformed by cube-root, PW by square-root and flake thickness by cube-root to achieve an approximately symmetrical distribution.

Full model: A Gaussian linear model summarizing the effect of PD, PW and EPA on flake thickness among the glass flake assemblage.

	Est. coef.	Std. error	z value	p
Intercept	1.47	0.01	119.61	<.001
PD	0.24	0.03	6.91	<.001
PW	-0.03	0.03	-0.76	0.45
EPA	0.01	0.01	0.93	0.35

F(3|146)=95.76, p<.001; Adjusted R^2=0.66.
Variance Inflation Factor: PD=7.81, PW=7.40, EPA=1.28.

Mediator model: A Gaussian linear model summarizing the effect of PD and EPA on PW among the glass flake assemblage.

	Est. coef.	Std. error	z value	p
Intercept	<0.0001	0.03	0.00	>0.99
PD	0.94	0.03	27.45	<.001

EPA	0.02	0.03	0.59	0.55

F(2|147)=470.7, p<.001; Adjusted R^2=0.86.
Variance Inflation Factor: PD=1.27, EPA=1.27.
