# Supplementary material for: The mediating effect of platform width on the size and shape of stone flakes
Source: PLoS One. 2022 Jan 21;17(1):e0262920. doi: 10.1371/journal.pone.0262920 (PMC8782408; doi:10.1371/journal.pone.0262920)
Supplement: S2 Table — (PDF) [file pone.0262920.s002.pdf]

Supplemental Material 2 Tables

SI Table 2a. Linear models used for the mediation analysis on flake weight among the flintknapped flake assemblage. PD is transformed by cube-root and PW by square-root to achieve an approximately symmetrical distribution.

**Full model**: A negative binomial linear model summarizing the effect of PD, PW and EPA on flake weight among the flintknapped flake assemblage.				
	Est. coef.	Std. error	z value	p
Intercept	2.38	0.04	56.70	<.001
PD	0.73	0.06	12.22	<.001
PW	0.33	0.04	7.90	<.001
EPA	0.31	0.06	5.18	<.001
Pearson-based dispersion value=1.45.<br>Likelihood ratio test: test statistic=333.28, df=3, p<.001.<br>Variance Inflation Factor: PD=1.91, PW=1.88, EPA=1.03.				
**Mediator model**: A Gaussian linear model summarizing the effect of PD and EPA on PW among the flintknapped flake assemblage.				
	Est. coef.	Std. error	z value	p
Intercept	<0.0001	0.03	0.00	>0.99
PD	0.72	0.03	21.81	<.001
EPA	-0.003	0.03	-0.09	0.93
$F(2\|461)=243.2$, $p<.001$; Adjusted $R^2=0.51$.<br>Variance Inflation Factor: PD=1.02, EPA=1.02.				

SI Table 2b. Linear models used for the mediation analysis on flake length among the flintknapped flake assemblage. PD is transformed by cube-root, PW by square-root and flake length by cube-root to achieve an approximately symmetrical distribution.

**Full model**: A Gaussian linear model summarizing the effect of PD, PW and EPA on flake length among the flintknapped flake assemblage.				
	Est. coef.	Std. error	z value	p
Intercept	3.44	0.02	163.51	<.001
PD	0.30	0.03	9.83	<.001
PW	-0.01	0.03	-0.39	0.70
EPA	0.13	0.02	5.97	<.001
$F(3\|460)=67.27$, $p<.001$; Adjusted $R^2=0.30$.<br>Variance Inflation Factor: PD=2.07, PW=2.05, EPA=1.02.				
**Mediator model**: A Gaussian linear model summarizing the effect of PD and EPA on PW among the flintknapped flake assemblage.				
	Est. coef.	Std. error	z value	p
Intercept	<0.0001	0.03	0.00	>0.99
PD	0.72	0.03	21.81	<.001
EPA	-0.003	0.03	-0.09	0.93

$F(2\|461)=243.2$, $p<.001$; Adjusted $R^2=0.51$.
Variance Inflation Factor: PD=1.02, EPA=1.02.

SI Table 2c. Linear models used for the mediation analysis on flake width among the flintknapped flake assemblage. PD is transformed by cube-root, PW by square-root and flake width by cube-root to achieve an approximately symmetrical distribution.

Full model: A Gaussian linear model summarizing the effect of PD, PW and EPA on flake width among the flintknapped flake assemblage.

	Est. coef.	Std. error	z value	p
Intercept	3.03	0.02	187.77	<.001
PD	0.11	0.02	4.59	<.001
PW	0.23	0.02	9.74	<.001
EPA	0.07	0.02	4.09	<.001

$F(3|460)=122.6$, $p<.001$; Adjusted $R^2=0.44$.
Variance Inflation Factor: PD=2.07, PW=2.05, EPA=1.02.

Mediator model: A Gaussian linear model summarizing the effect of PD and EPA on PW among the flintknapped flake assemblage.

	Est. coef.	Std. error	z value	p
Intercept	<0.0001	0.03	0.00	>0.99
PD	0.72	0.03	21.81	<.001
EPA	-0.003	0.03	-0.09	0.93

$F(2|461)=243.2$, $p<.001$; Adjusted $R^2=0.51$.
Variance Inflation Factor: PD=1.02, EPA=1.02.

SI Table 2d. Linear models used for the mediation analysis on flake thickness among the flintknapped flake assemblage. PD is transformed by cube-root, PW by square-root and flake thickness by cube-root to achieve an approximately symmetrical distribution.

Full model: A Gaussian linear model summarizing the effect of PD, PW and EPA on flake thickness among the flintknapped flake assemblage.

	Est. coef.	Std. error	z value	p
Intercept	1.75	0.01	128.80	<.001
PD	0.25	0.02	12.55	<.001
PW	0.06	0.02	3.12	0.002
EPA	0.06	0.01	4.44	<.001

$F(3|460)=151.8$, $p<.001$; Adjusted $R^2=0.49$.
Variance Inflation Factor: PD=2.07, PW=2.05, EPA=1.02.

Mediator model: A Gaussian linear model summarizing the effect of PD and EPA on PW among the flintknapped flake assemblage.

	Est. coef.	Std. error	z value	p
Intercept	<0.0001	0.03	0.00	>0.99
PD	0.72	0.03	21.81	<.001

EPA	-0.003	0.03	-0.09	0.93

F(2|461)=243.2, $p<.001$; Adjusted R^2=0.51.
Variance Inflation Factor: PD=1.02, EPA=1.02.
