# Supplementary material for: The mediating effect of platform width on the size and shape of stone flakes
Source: PLoS One. 2022 Jan 21;17(1):e0262920. doi: 10.1371/journal.pone.0262920 (PMC8782408; doi:10.1371/journal.pone.0262920)

The mediating effect of platform width on the size and shape of stone flakes

Sam Lin

31 May 2021

This rMarkdown file contains the code used to produce the statistical results described in the paper. Note that there will be minor variation in the coefficients of the mediation analyses due to the quasi-Bayesian Monte Carlo simulation. These variation do not impact the finding of the study.

Fig 5. Boxplot summarizing the distribution of the PW-PD ratio among the glass flakes by platform profile type.

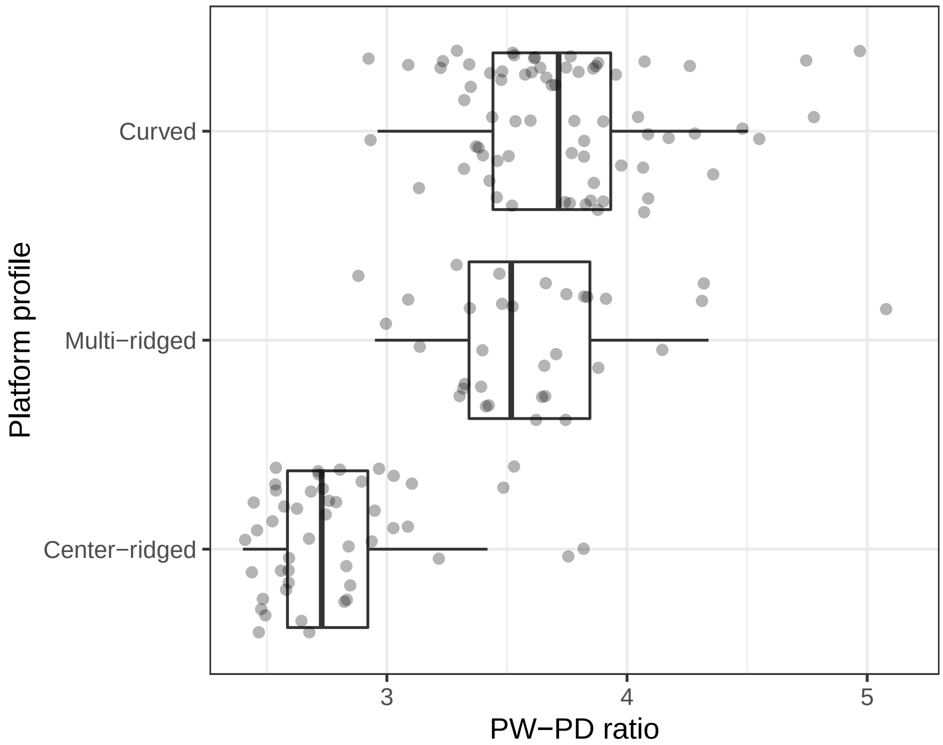

ANOVA test of the PW-PD ratio by the three platform profile types among the glass flakes.

```
##              Df Sum Sq Mean Sq F value Pr(>F)
## plattype      2  2.060  1.0298   99.41 <2e-16 ***
## Residuals   147  1.523  0.0104
## ---
## Signif. codes:  0 '***' 0.001 '**' 0.01 '*' 0.05 '.' 0.1 ' ' 1
```

```
##   Tukey multiple comparisons of means
##     95% family-wise confidence level
##
## Fit: aov(formula = sqrt(PW_PD) ~ plattype, data = glassdata)
##
## $plattype
##                                   diff         lwr         upr     p adj
## Multi-ridged-Curved        -0.03454003 -0.08596313  0.01688307 0.2530153
## Center-ridged-Curved       -0.26043316 -0.30559311 -0.21527320 0.0000000
## Center-ridged-Multi-ridged -0.22589313 -0.28088918 -0.17089707 0.0000000
```

Information in Fig 6. The mediating effect of PW on the causal influence of PD and EPA on flake attributes among the glass flake assemblage.

Flake weight

The mediating effect of PW on the causal influence of PD on flake weight among the glass flake assemblage.

```
##
## Causal Mediation Analysis
##
## Nonparametric Bootstrap Confidence Intervals with the Percentile Method
##
##                          Estimate 95% CI Lower 95% CI Upper p-value
## ACME (control)              5.491        3.595         7.48  <2e-16 ***
## ACME (treated)             10.294        7.267        13.21  <2e-16 ***
## ADE (control)               8.666        6.409        11.09  <2e-16 ***
## ADE (treated)              13.469       10.668        16.43  <2e-16 ***
## Total Effect               18.960       15.867        22.38  <2e-16 ***
## Prop. Mediated (control)    0.290        0.202         0.38  <2e-16 ***
## Prop. Mediated (treated)    0.543        0.424         0.65  <2e-16 ***
## ACME (average)              7.892        5.442        10.26  <2e-16 ***
## ADE (average)              11.068        8.581        13.68  <2e-16 ***
## Prop. Mediated (average)    0.416        0.313         0.51  <2e-16 ***
## ---
## Signif. codes:  0 '***' 0.001 '**' 0.01 '*' 0.05 '.' 0.1 ' ' 1
##
## Sample Size Used: 150
##
##
## Simulations: 10000
```

The mediating effect of PW on the causal influence of EPA on flake weight among the glass flake assemblage.

```
##
## Causal Mediation Analysis
##
## Nonparametric Bootstrap Confidence Intervals with the Percentile Method
##
##                          Estimate 95% CI Lower 95% CI Upper p-value
## ACME (control)             0.1424      -0.2952         0.63    0.53
## ACME (treated)             0.2552      -0.5251         1.15    0.53
## ADE (control)             11.7860       8.9999        15.52  <2e-16 ***
## ADE (treated)             11.8989       9.0345        15.73  <2e-16 ***
## Total Effect              12.0412       9.0594        16.02  <2e-16 ***
## Prop. Mediated (control)   0.0118      -0.0268         0.05    0.53
## Prop. Mediated (treated)   0.0212      -0.0483         0.09    0.53
## ACME (average)             0.1988      -0.4085         0.89    0.53
## ADE (average)             11.8424       9.0164        15.64  <2e-16 ***
## Prop. Mediated (average)   0.0165      -0.0377         0.07    0.53
```

```
## ---
## Signif. codes:  0 '***' 0.001 '**' 0.01 '*' 0.05 '.' 0.1 ' ' 1
##
## Sample Size Used: 150
##
##
## Simulations: 10000
```

Flake length

The mediating effect of PW on the causal influence of PD on flake length among the glass flake assemblage.

```
##
## Causal Mediation Analysis
##
## Nonparametric Bootstrap Confidence Intervals with the Percentile Method
##
##                Estimate 95% CI Lower 95% CI Upper p-value
## ACME             -1.391       -9.385         5.83    0.69
## ADE              20.182       12.416        28.81  <2e-16 ***
## Total Effect     18.791       15.951        21.62  <2e-16 ***
## Prop. Mediated   -0.074       -0.505         0.31    0.69
## ---
## Signif. codes:  0 '***' 0.001 '**' 0.01 '*' 0.05 '.' 0.1 ' ' 1
##
## Sample Size Used: 150
##
##
## Simulations: 10000
```

The mediating effect of PW on the causal influence of EPA on flake length among the glass flake assemblage.

```
##
## Causal Mediation Analysis
##
## Nonparametric Bootstrap Confidence Intervals with the Percentile Method
##
##                Estimate 95% CI Lower 95% CI Upper p-value
## ACME           -0.03007     -0.41571         0.31    0.88
## ADE            19.81948     17.58936        22.11  <2e-16 ***
## Total Effect   19.78942     17.58446        22.09  <2e-16 ***
## Prop. Mediated -0.00152     -0.02121         0.02    0.88
## ---
## Signif. codes:  0 '***' 0.001 '**' 0.01 '*' 0.05 '.' 0.1 ' ' 1
##
## Sample Size Used: 150
##
##
## Simulations: 10000
```

Flake width

The mediating effect of PW on the causal influence of PD on flake width among the glass flake assemblage.

```
##
## Causal Mediation Analysis
##
## Nonparametric Bootstrap Confidence Intervals with the Percentile Method
##
##                 Estimate 95% CI Lower 95% CI Upper p-value
## ACME               9.727        6.232        13.35  <2e-16 ***
## ADE               -3.110       -6.774         0.53    0.09 .
## Total Effect       6.617        5.328         7.96  <2e-16 ***
## Prop. Mediated     1.470        0.925         2.11  <2e-16 ***
## ---
## Signif. codes:  0 '***' 0.001 '**' 0.01 '*' 0.05 '.' 0.1 ' ' 1
##
## Sample Size Used: 150
##
##
## Simulations: 10000
```

The mediating effect of PW on the causal influence of EPA on flake width among the glass flake assemblage.

```
##
## Causal Mediation Analysis
##
## Nonparametric Bootstrap Confidence Intervals with the Percentile Method
##
##                 Estimate 95% CI Lower 95% CI Upper p-value
## ACME               0.210       -0.440         0.92  0.5428
## ADE                1.892        1.114         2.83  <2e-16 ***
## Total Effect       2.102        1.033         3.31  0.0006 ***
## Prop. Mediated     0.100       -0.348         0.36  0.5422
## ---
## Signif. codes:  0 '***' 0.001 '**' 0.01 '*' 0.05 '.' 0.1 ' ' 1
##
## Sample Size Used: 150
##
##
## Simulations: 10000
```

Flake thickness

The mediating effect of PW on the causal influence of PD on flake thickness among the glass flake assemblage.

```
##
## Causal Mediation Analysis
##
## Nonparametric Bootstrap Confidence Intervals with the Percentile Method
##
##                 Estimate 95% CI Lower 95% CI Upper p-value
## ACME             -0.0240      -0.0791         0.03    0.39
## ADE               0.2398       0.1705         0.31  <2e-16 ***
## Total Effect      0.2158       0.1849         0.25  <2e-16 ***
## Prop. Mediated   -0.1113      -0.3669         0.16    0.39
## ---
## Signif. codes:  0 '***' 0.001 '**' 0.01 '*' 0.05 '.' 0.1 ' ' 1
##
## Sample Size Used: 150
##
##
## Simulations: 10000
```

The mediating effect of PW on the causal influence of EPA on flake thickness among the glass flake assemblage.

```
##
## Causal Mediation Analysis
##
## Nonparametric Bootstrap Confidence Intervals with the Percentile Method
##
##                  Estimate 95% CI Lower 95% CI Upper p-value
## ACME           -0.000519    -0.004123         0.00    0.73
## ADE             0.013112    -0.011772         0.04    0.31
## Total Effect    0.012592    -0.012251         0.04    0.33
## Prop. Mediated -0.041233    -0.855256         0.68    0.82
##
## Sample Size Used: 150
##
##
## Simulations: 10000
```

Information in Fig 7. The mediating effect of PW on the causal influence of PD and EPA on flake attributes among the flintknapped flake assemblage.

Flake weight

The mediating effect of PW on the causal influence of PD on flake weight among the flintknapped flake assemblage.

```
##
## Causal Mediation Analysis
##
## Nonparametric Bootstrap Confidence Intervals with the Percentile Method
##
##                          Estimate 95% CI Lower 95% CI Upper p-value
## ACME (control)             2.8624       1.4839         4.58  <2e-16 ***
## ACME (treated)             5.9132       3.3367         8.72  <2e-16 ***
## ADE (control)             12.4860       9.4106        15.98  <2e-16 ***
## ADE (treated)             15.5369      12.4079        18.96  <2e-16 ***
## Total Effect              18.3993      15.3227        21.86  <2e-16 ***
## Prop. Mediated (control)   0.1556       0.0826         0.25  <2e-16 ***
## Prop. Mediated (treated)   0.3214       0.1873         0.45  <2e-16 ***
## ACME (average)             4.3878       2.4189         6.62  <2e-16 ***
## ADE (average)             14.0115      10.9741        17.42  <2e-16 ***
## Prop. Mediated (average)   0.2385       0.1348         0.35  <2e-16 ***
## ---
## Signif. codes:  0 '***' 0.001 '**' 0.01 '*' 0.05 '.' 0.1 ' ' 1
##
## Sample Size Used: 464
##
##
## Simulations: 10000
```

The mediating effect of PW on the causal influence of EPA on flake weight among the flintknapped flake assemblage.

```
##
## Causal Mediation Analysis
##
## Nonparametric Bootstrap Confidence Intervals with the Percentile Method
##
##                          Estimate 95% CI Lower 95% CI Upper p-value
## ACME (control)           -0.01628     -0.61805         0.48    0.89
## ACME (treated)           -0.02263     -0.87631         0.66    0.89
## ADE (control)             6.78025      3.75548        10.57  <2e-16 ***
## ADE (treated)             6.77391      3.76593        10.48  <2e-16 ***
## Total Effect              6.75762      3.73455        10.39  <2e-16 ***
## Prop. Mediated (control) -0.00241     -0.10331         0.08    0.89
## Prop. Mediated (treated) -0.00335     -0.14132         0.10    0.89
## ACME (average)           -0.01946     -0.74460         0.57    0.89
## ADE (average)             6.77708      3.77016        10.52  <2e-16 ***
## Prop. Mediated (average) -0.00288     -0.12112         0.09    0.89
```

```
## ---
## Signif. codes:  0 '***' 0.001 '**' 0.01 '*' 0.05 '.' 0.1 ' ' 1
##
## Sample Size Used: 464
##
##
## Simulations: 10000
```

Flake length

The mediating effect of PW on the causal influence of PD on flake length among the flintknapped flake assemblage.

```
##
## Causal Mediation Analysis
##
## Nonparametric Bootstrap Confidence Intervals with the Percentile Method
##
##                 Estimate 95% CI Lower 95% CI Upper p-value
## ACME           -0.00846     -0.05316         0.03    0.67
## ADE             0.29854      0.24421         0.36  <2e-16 ***
## Total Effect    0.29008      0.25226         0.33  <2e-16 ***
## Prop. Mediated -0.02916     -0.18706         0.11    0.67
## ---
## Signif. codes:  0 '***' 0.001 '**' 0.01 '*' 0.05 '.' 0.1 ' ' 1
##
## Sample Size Used: 464
##
##
## Simulations: 10000
```

The mediating effect of PW on the causal influence of EPA on flake length among the flintknapped flake assemblage.

```
##
## Causal Mediation Analysis
##
## Nonparametric Bootstrap Confidence Intervals with the Percentile Method
##
##                 Estimate 95% CI Lower 95% CI Upper p-value
## ACME            3.62e-05    -3.11e-03         0.00    0.94
## ADE             1.27e-01     8.76e-02         0.17  <2e-16 ***
## Total Effect    1.27e-01     8.81e-02         0.17  <2e-16 ***
## Prop. Mediated  2.85e-04    -2.41e-02         0.03    0.94
## ---
## Signif. codes:  0 '***' 0.001 '**' 0.01 '*' 0.05 '.' 0.1 ' ' 1
##
## Sample Size Used: 464
##
##
## Simulations: 10000
```

Flake width

The mediating effect of PW on the causal influence of PD on flake width among the flintknapped flake assemblage.

```
##
## Causal Mediation Analysis
##
## Nonparametric Bootstrap Confidence Intervals with the Percentile Method
##
##                 Estimate 95% CI Lower 95% CI Upper p-value
## ACME              0.1613       0.1239         0.21  <2e-16 ***
## ADE               0.1068       0.0523         0.15  <2e-16 ***
## Total Effect      0.2681       0.2371         0.30  <2e-16 ***
## Prop. Mediated    0.6017       0.4566         0.79  <2e-16 ***
## ---
## Signif. codes:  0 '***' 0.001 '**' 0.01 '*' 0.05 '.' 0.1 ' ' 1
##
## Sample Size Used: 464
##
##
## Simulations: 10000
```

The mediating effect of PW on the causal influence of EPA on flake width among the flintknapped flake assemblage.

```
##
## Causal Mediation Analysis
##
## Nonparametric Bootstrap Confidence Intervals with the Percentile Method
##
##                  Estimate 95% CI Lower 95% CI Upper p-value
## ACME           -0.000691    -0.023896         0.02  0.9108
## ADE             0.066630     0.032539         0.11  0.0002 ***
## Total Effect    0.065939     0.028774         0.11  0.0004 ***
## Prop. Mediated -0.010479    -0.551928         0.27  0.9112
## ---
## Signif. codes:  0 '***' 0.001 '**' 0.01 '*' 0.05 '.' 0.1 ' ' 1
##
## Sample Size Used: 464
##
##
## Simulations: 10000
```

Flake thickness

The mediating effect of PW on the causal influence of PD on flake thickness among the flintknapped flake assemblage.

```
##
## Causal Mediation Analysis
##
## Nonparametric Bootstrap Confidence Intervals with the Percentile Method
##
##                Estimate 95% CI Lower 95% CI Upper p-value
## ACME             0.0435       0.0156         0.07   0.001 ***
## ADE              0.2455       0.2079         0.28  <2e-16 ***
## Total Effect     0.2890       0.2647         0.31  <2e-16 ***
## Prop. Mediated   0.1505       0.0543         0.25   0.001 ***
## ---
## Signif. codes:  0 '***' 0.001 '**' 0.01 '*' 0.05 '.' 0.1 ' ' 1
##
## Sample Size Used: 464
##
##
## Simulations: 10000
```

The mediating effect of PW on the causal influence of EPA on flake thickness among the flintknapped flake assemblage.

```
##
## Causal Mediation Analysis
##
## Nonparametric Bootstrap Confidence Intervals with the Percentile Method
##
##                 Estimate 95% CI Lower 95% CI Upper p-value
## ACME           -0.000186    -0.006848         0.01     0.9
## ADE             0.060949     0.033118         0.09  <2e-16 ***
## Total Effect    0.060763     0.032645         0.09  <2e-16 ***
## Prop. Mediated -0.003066    -0.130617         0.10     0.9
## ---
## Signif. codes:  0 '***' 0.001 '**' 0.01 '*' 0.05 '.' 0.1 ' ' 1
##
## Sample Size Used: 464
##
##
## Simulations: 10000
```

Pearson's correlation between the PW-PD ratio and the blank surface area to thickness ratio and the elongation ratio respectively among the flintknapped assemblage.

PW-PD ratio vs. blank surface area to thickness ratio

```
##
##  Pearson's product-moment correlation
##
## data:  flintdata$PW_PD and flintdata$SA_thickness
## t = 4.3533, df = 462, p-value = 1.652e-05
## alternative hypothesis: true correlation is not equal to 0
## 95 percent confidence interval:
##  0.109450 0.284397
## sample estimates:
##       cor
## 0.1985042
```

PW-PD ratio vs. elongation ratio

```
##
##  Pearson's product-moment correlation
##
## data:  flintdata$PW_PD and flintdata$elongation
## t = -5.4076, df = 462, p-value = 1.027e-07
## alternative hypothesis: true correlation is not equal to 0
## 95 percent confidence interval:
##  -0.3277325 -0.1564217
## sample estimates:
##        cor
## -0.2439795
```

Fig 8. The distribution of the PW-PD ratio, the elongation ratio and the flake surface area to thickness ratio by platform profile type among the flake assemblage from Roc de Marsal (Dordogne, France). Only complete flakes are included in the analysis. For the analysis of variance (ANOVA), all three variables were transformed to achieve an approximately symmetrical distribution to meet the assumption of the test.

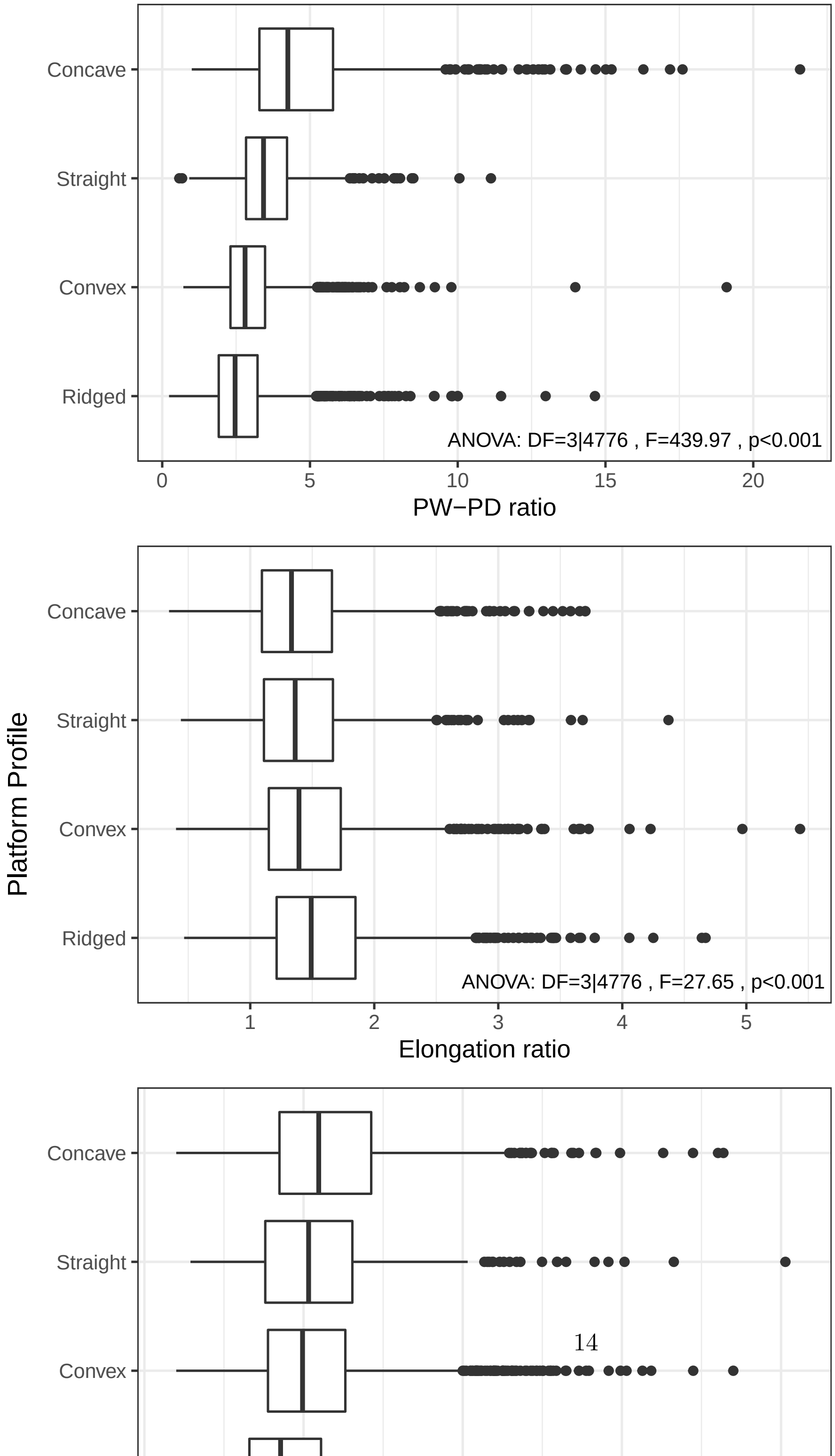

Supplement: S1 File — (ZIP) [file pone.0262920.s003.zip › Lin_et_al_PW_mediation.pdf]
