# Supplementary material for: The mediating effect of platform width on the size and shape of stone flakes
Source: PLoS One. 2022 Jan 21;17(1):e0262920. doi: 10.1371/journal.pone.0262920 (PMC8782408; doi:10.1371/journal.pone.0262920)
Supplement: Attachment — Submitted filename: Response to reviewers.docx [file pone.0262920.s004.docx]

**Editor**

| *Comment* | *Response* |
| --- | --- |
| Please ensure the manuscript meets PLOS ONE style requirements. | - Removed numbering in headings within the manuscript. - Corrected the naming of the figure files. - Figures have been corrected using PACE. |
| Please provide additional information regarding the specimens used in your study. | - No permits were required for this study. The relevant statement of declaration is outlined in lines 367-368. - Information about the current locations of the studied experimental datasets have been added into the Material and Methods section (lines 228-230 and 320-321). |
| Please ensure that you provide the correct grant numbers for the awards you received for your study in the ‘Funding Information’ section. | - ‘Funding Information’ section updated to match the information provided in ‘Financial Disclosure’. |
| Please review your reference list to ensure that it is complete and correct. | - All references reviewed. Minor corrections in error and formatting. |

**Reviewer 1**

| *Comment* | *Response* |
| --- | --- |
| My bias is that I would question the overall approach of using such correlations to investigate the process of brittle fracture which is a well developed branch of physics. | We have included additional text in Introduction (lines 71-75) and Material and Methods (lines 253-259) to explain why we have taken a correlation approach here instead of using fracture mechanic models. |
| I would suggest that they first clearly delineate their model for fracture mechanics and then directly link this to the hypotheses that lead to the analysis… The model of fracture dynamics are in a sense the most significant part of the paper so this should be highlighted. | We have added text in Introduction (lines 179-184) to state our model of flake formation before outlining the specific hypotheses tested in this study. |
| I was particularly confused on line 273 when we seemingly are told a new hypothesis that was going to be tested. | This paragraph in Material and Methods has been removed. |
| …why do we wait to near the end of the paper to get a section on 'a new model'-- shouldn't this be up front as the model is not derived from the data but rather is the source of the hypotheses that are tested? | Text has been added in Introduction to explicitly state our hypothesised model up front (lines 179-184). The Discussion has been modified to make it clear that the section on the fracture model is an reiteration of our hypothesised model. The subheading of “A New Model of Fracture Initiation and Propagation” has been removed to avoid confusion. |
| …there does need to be an explanation of why the authors do not use a statistical method used normally by physicists studying fracture mechanics. From the text it appears that this is a social sciences method and it is really unclear why it is being used in a materials science analysis… There needs to be a clear discussion of alternative statistical approaches available for study fracture mechanics and why mediation analysis was chosen as most appropriate. | Text has been added to Material and Methods to explain why we employed a mediation analysis here (lines 259-262; 305-310). We have also included a new paragraph to provide more background information about mediation analysis, showing that the approach has been applied in fields beyond the social sciences. |
| Line 67-69—seems a great overstatement to say that lithic manufacture has become synonymous with lithic technology and the role of replicative studies is exaggerated. | This statement has been removed. |
| Line 77—not clear what platform setting refers to. | Reworded to ‘the shape of the platform surface.’ |
| Line 84-87—Again seems to be an exaggeration, makes it sound that shifts in EPA or PD control flake morphology independent of other factors. Thus in line 96-100 the authors contradict their earlier statement. | We have modified the sentence in Line 86-87 so that it only summarises the experimental relationship. |
| Line 143—Arrangement is the wrong word here. | Reworded to ‘relationship.’ |

**Reviewer 2**

| *Comment* | *Response* |
| --- | --- |
| The PW-PD ratios appear non-normally distributed (especially in fig8), for which parametric tests like ANOVA and Tukey are ill suited. It is likely that a Wilcoxon rank-sum test and post-hoc Kruskal-Wallis tests are more appropriate. | We have specified in our original text that all variables were transformed to achieve an approximately symmetrical distribution to fulfil the assumption of the statistical tests used (e.g., lines 377-378 and the caption for Fig 8). |
| …some more mention could be made of the broader significance of understanding platform-flake relationships. | Additional text has been added in Discussions (lines 502-506) to relate the study results to broader topics of lithic technology. |
| L68: ‘very much’ can be deleted or replaced with something like ‘considerably’ | Removed. |
| L331: ‘casual’ should be ‘causal’, you may want to do a find and replace, Word may have autocorrected this a few times. | Corrected throughout the text. |
