# Supplementary material for: The mediating effect of platform width on the size and shape of stone flakes
Source: PLoS One. 2022 Jan 21;17(1):e0262920. doi: 10.1371/journal.pone.0262920 (PMC8782408; doi:10.1371/journal.pone.0262920)
Supplement: Attachment — Submitted filename: Response to Reviewers.docx [file pone.0262920.s005.docx]

**Reviewer 1 comment:**

The fundamental issue with this paper is that it claims to be finding causal relationships between variables based on correlations. Thus the authors argue that they are making fundamental observations about fracture mechanics while not relying on any methods from the study of fracture mechanics.

My sense is that the authors’ have not quite identified what it is that they are actually doing in their study. They have definitively not determined a chain of causal factors controlling fracture in brittle solids.

I think what interests these authors, and what interests me as well, is not the actual physics of fracture mechanics but rather the visually accessible attributes that would be available to a knapper attempting to control the attributes of knapped flakes. Thus their work is focused not on the fracture mechanics themselves (a process that takes place at the atomic and molecular scale) but rather the cues that would be available to a knapper in the process of flake production. In that framework the data presented here makes sense as the causal chain would relate to the knapper’s inference of a causal chain of related attributes.

My sense is that they are trying to get at something, and have produced relevant data, that is important but that they have not clarified what their goals actually are.

**Response**:

We agree with the reviewer that our study is concerned about the relationship between knapping factors and flaking outcome, rather than the physical processes of fracture mechanics themselves. We have included additional text in the manuscript to make this distinction clear. For example, after outlining our proposed conceptual model of platform formation in Introduction, we added the following text (lines 191-194):

“Undoubtedly, this model is simplistic and does not represent the actual mechanisms underlying the fracture process. However, as a conceptual heuristic, the model can help us hypothesize about the interrelationship between platform variables, such as PD and PW, and flake attributes.”

A similar section is now included Material & Methods (lines 265-273):

“We acknowledge that this approach does not investigate the actual causal mechanisms underlying the fracture process – such mechanisms will need to be understood from principles of fracture mechanics. Instead, we are interested in characterizing the relationships among the flake variables in question here through statistical modeling... Note that terms such as ‘effect’, ‘influence’ and ‘impact’ are used here strictly to describe statistical relationships among the independent and dependent test variables, rather than the underlying causal mechanisms of fracture.”

We have also made changes throughout the manuscript to make clear the distinction thtat we are mainly discussing statistical relationships rather than the causal mechanisms of flake fracture. These changes mainly occurred in Results and Discussion.
